# Scandium Decoration of Boron Doped Porous Graphene for High-Capacity Hydrogen Storage

**DOI:** 10.3390/molecules24132382

**Published:** 2019-06-27

**Authors:** Jing Wang, Yuhong Chen, Lihua Yuan, Meiling Zhang, Cairong Zhang

**Affiliations:** 1 State Key Laboratory of Advanced Processing and Recycling of Non-Ferrous Metals, Lanzhou University of Technology, Lanzhou 730050, China; 2 School of Science, Lanzhou University of Technology, Lanzhou 730050, China

**Keywords:** first principles, porous graphene, B doped Sc modified PG, hydrogen storage

## Abstract

The hydrogen storage properties of the Scandium (Sc) atom modified Boron (B) doped porous graphene (PG) system were studied based on the density functional theory (DFT). For a single Sc atom, the most stable adsorption position on B-PG is the boron-carbon hexagon center after doping with the B atom. The corresponding adsorption energy of Sc atoms was −4.004 eV. Meanwhile, five H_2_ molecules could be adsorbed around a Sc atom with the average adsorption energy of −0.515 eV/H_2_. Analyzing the density of states (DOS) and the charge population of the system, the adsorption of H_2_ molecules in Sc-B/PG system is mainly attributed to an orbital interaction between H and Sc atoms. For the H_2_ adsorption, the Coulomb attraction between H_2_ molecules (negatively charged) and Sc atoms (positively charged) also played a critical role. The largest hydrogen storage capacity structure was two Sc atoms located at two sides of the boron-carbon hexagon center in the Sc-B/PG system. Notably, the theoretical hydrogen storage capacity was 9.13 wt.% with an average adsorption energy of −0.225 eV/H_2_. B doped PG prevents the Sc atom aggregating and improves the hydrogen storage effectively because it can increase the adsorption energy of the Sc atom and H_2_ molecule.

## 1. Introduction

The development of human society faces the severe challenge of environmental pollution, which is urgently needed to explore more ideal energy materials to meet the growing energy demand [1,2,3]. Hydrogen (H_2_) is used as a promising energy carrier because of recycling, no pollution, high energy density and high calorific value [4,5,6]. Graphene is a two-dimensional material with a hexagonal honeycomb structure composed of sp2 hybrid orbits. Graphene-based materials possess large specific surface area, good adsorption kinetics, low density, high chemical stability and reversible hydrogen storage, which is a potential candidate in many areas, such as solid-state hydrogen storage, electronics and sensors [7,8,9,10,11,12,13,14]. However, due to a large surface inertness and weak binding ability to H_2_ molecules, clean graphene is difficult to become a promising hydrogen storage material [15,16,17,18,19]. 

Recently, transition metal atoms [20,21] modified graphene has attracted researchers’ attention since significantly increasing the adsorption energy of H_2_ molecules. Faye et al. [22] found that Pd double-sided modified graphene could adsorb up to eight H_2_ molecules using local density approximation (LDA) of Dmol3 software. The transition metal atoms Sc, Ti and V decorated on both sides of graphene could adsorb four H_2_ molecules with an average adsorption energy of 0.300–0.500 eV/H_2_ [23].

Yet the transition metal atom modified graphene is prone to aggregation, which reduces the adsorption site of H_2_ and weakens the hydrogen storage performance of graphene. Investigations have shown that the introduction of impurity atoms or vacancies can prevent the agglomeration of transition metal atoms and increase the hydrogen storage capacity of graphene [24]. There are two effective means available at present. The first method is a non-metal atom such as B, N etc. doped graphene [25,26,27,28]. Since the B atom has an empty p orbit, it can be used as an electron acceptor to obtain the electrons of other modified atoms, enhancing the adsorption energy of the modified atom [29]. Meanwhile, B-doped graphene has been successfully synthesized in experiments [30]. To introduce pore defects into graphene is another method [31,32]. Compared with non-defective graphene, there is no agglomeration between the metals because the metal atoms are closer to the substrate with a high metal binding energy. Moreover, the porous material adsorbs H_2_ through Van der Waals (VDW) interactions between the system and the H_2_ molecule, which is physical adsorption. Porous graphene (PG) [33] is a two-dimensional material synthesized in experiments with high specific surface area, which can store hydrogen efficiently [34]. Du [2] used local density approximation (LDA) of the VASP code to study the adsorption of H_2_ on the Li atom modified porous graphene. The hydrogen storage capacity of the system can reach 12.00 wt.%, and the average adsorption energy is about 0.243 eV. However, the LDA functional will overestimate the average binding energy of H_2_ molecules. Additionally, Reunchan [35] and Yuan [36] studied the hydrogen storage properties of Ca and Y atom-modified porous graphene by generalized gradient approximation (GGA) functional theory.

The research found that the combination of these two methods could avoid the aggregation of transition metal atoms on the PG surface, meet the requirement of the U.S. Department of Energy (DOE) for hydrogen storage, but also make the adsorption energy of H_2_ molecules in the range of reversible hydrogen storage [37,38]. Simultaneously, Sc modified porous graphene possessed a larger amount of hydrogen storage because of the lightest atomic mass in the transition metal atom. However, there are only a few reports on the adsorption mechanism and hydrogen storage capacity of Sc decorated B-doped PG. 

Herein, we combined the Sc modification and the B doping methods to modify the porous graphene. In this paper, the most stable adsorption structure and adsorption energy of Sc decorated B-doped PG were manipulated via a first-principles calculation. In addition to this, the performance and mechanism of the H_2_ molecule adsorption in Sc-modified B-PG system were analyzed deeply. Based on the results, the largest hydrogen storage capacity structure was two Sc atoms located at two sides of the boron-carbon hexagon center in the Sc-B/PG system. Theoretically, the hydrogen storage capacity was 9.13 wt.% with an average adsorption energy of −0.225 eV/H_2_. B doped PG prevents the Sc atom aggregating and improves the hydrogen storage effectively because it can increase the adsorption energy of the Sc atom and H_2_ molecule.

## 2. Calculation Details

Based on the density functional theory, the GGA function of the CASTEP software package [39] was used to select the PBE exchange correlation gradient correction [40]. The Van der Waals correction (DFT-D method) was used in order to avoid the GGA function underestimation of energy in the calculation. All the atoms in the calculation were completely relaxed. The convergence criteria of the optimized structure were as follows: Each atom was subjected to a force below 0.01 eV/Å; the energy convergence tolerance was lower than 5.0 × $10^{-6}$ eV/atom; the self-consistent field convergence threshold was selected as 1.0 ×$\;10^{-6}$ eV/atom between two steps. The vacuum layer was selected as 20 Å along the Z direction of the PG to eliminate the interlayer interaction. Considering the calculation accuracy and calculation cost, the cutoff energy was selected as 500 eV, and the K-point in the Brillouin zone was 7 × 7 × 1.

Formula (1) is used to define the average binding energy (${\bar{E}}_{b}$) of Sc atoms on B-PG:

(1) $${\bar{E}}_{b\;}=\;[E_{Sc+B/PG}-E_{B/PG}-nE_{Sc}]/n$$

among which $E_{Sc+B/PG}\;$,$\;E_{B/PG}$ and $E_{Sc}$ represent the energy in the Sc-modified B-doped PG system, the B-doped PG system and a free Sc atom, respectively. Additionally, n represents the number of adsorbed Sc atoms.

The adsorption energy ($E_{ad}$) and average adsorption energy (${\bar{E}}_{ad}$) of the H_2_ molecule is defined as:

(2) $$E_{ad}\;=\;\left[E_{iH_{2}+Sc+B/PG}-E_{\left(i-1\right)H_{2}+Sc+B/PG}-E_{H_{2}}\right]$$

(3) $${\bar{E}}_{ad}\;=\;\left[E_{iH_{2}+Sc+B/PG}-E_{Sc+B/PG}-iE_{H_{2}}\right]/i$$

where $E_{iH_{2}+Sc+B/PG}$, $E_{\left(i-1\right)H_{2}+Sc+B/PG}$ and $E_{H_{2}}$ respectively represent the total energy of the system with i, (i-1) H_2_ molecules adsorbed and the total energy of a free H_2_ molecule.

The formation energy [41] is defined as:

(4) $$E_{f}\;=\;\left[E_{B/PG}-n_{C}\mu_{C}-n_{H}\mu_{H}-n_{B}\mu_{B}\right]/\left[n_{C}+n_{H}+n_{B}\right]$$

where $E_{B/PG}$ is the energy of the doped porous graphene, $n_{C},\;n_{H}$ and $n_{B}$ are the number of C, H and B atoms in the doped porous graphene, respectively. $\mu_{C}$, $\mu_{H}$, $\mu_{B}$ denote the chemical potential of C, H, B atoms, respectively ($\mu_{C}=E_{graphene}/$number of carbon atoms in graphene, $\mu_{H}=E_{H_{2}}/$2, $\mu_{B}=E_{B_{12}crystal}/$12).

## 3. Results and Discussion

### 3.1. Single B Atom Doped Stable Position

Firstly, six structures of B doped PG were considered. The optimized structures and lattice parameters of 1~6 B-doped PG systems are shown in Table 1. The calculation results show that: As the number of doped B atoms increased, the formation of the doping system became larger and larger, and the energy barrier to be overcome by the doping increased, making doping difficult, which was consistent with Lu et al. [38]. Therefore, we only considered the case of doping one B atom. The optimized structure of a single B-doped PG system is shown in Table 1. The lattice constant was 7.57 Å, the B-H bond length was 1.19 Å, and the formation energy required for doping was 121.3 meV/atom. The lattice constant of 7.57 Å was significantly higher than that of clean porous graphene 7.49 Å [37], but it was consistent with the lattice constant of 7.57 Å obtained by Lu [38]. The increase in the lattice constant was due to the radius of the B atom being greater than the radius of the replaced C atom. The measured B-H bond length of 1.19 Å was completely consistent with that of Lu et al. [38]. The formation energy of the B-PG doping system was 121.30 meV/atom, which was also close to the result of 120.20 meV/atom [38], indicating that the calculation method and accuracy were feasible.

**Figure 1:**
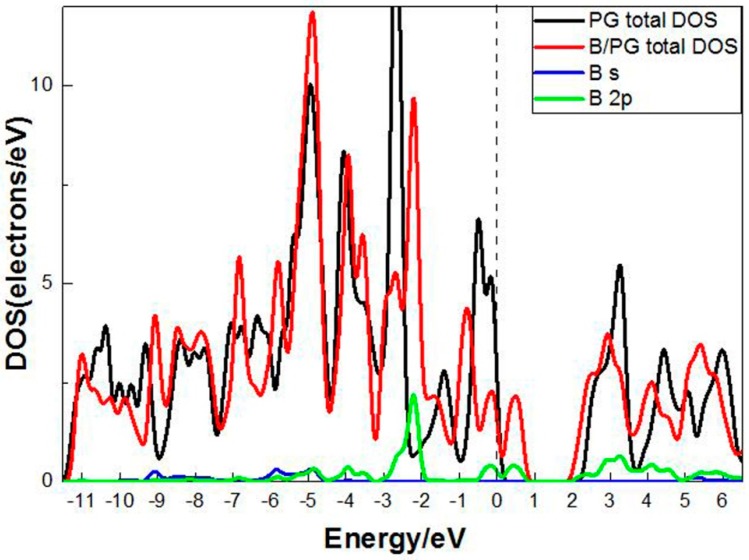
DOS of the porous graphene system with and without B-doped.

In order to study the effect of B atom doping on the performance of porous graphene, the density of states of a single B atom doped porous graphene system was calculated. The results are shown in Figure 1. It can be seen that there was an energy band passing near the Fermi surface after doping B atoms compared with the clean porous graphene density of states; the change of density of states near the Fermi surface of the doping system mainly came from the contribution of the 2p orbit of the B atom. In addition, the 2p orbit of the B atom overlapped with the porous graphene in the vicinity of the Fermi surface. We obtained the B-PG system band gap of 0.349 eV, which was much smaller than the undoped PG’s band gap value of 2.399 eV. From Figure 1, in the interval of −3.0~−2.0 eV, the peak of PG shifted to the right due to the influence of the B atom 2p orbit. The doping system electrons were close to the Fermi level. This indicates that B atom doping increases the chemical activity of the PG system to some extent and can improve the hydrogen storage performance of the PG system.

### 3.2. Single Sc Atom Modified B-PG System

#### 3.2.1. Adsorption Structure of Single Sc Atom Modified B-PG

**Figure 2:**
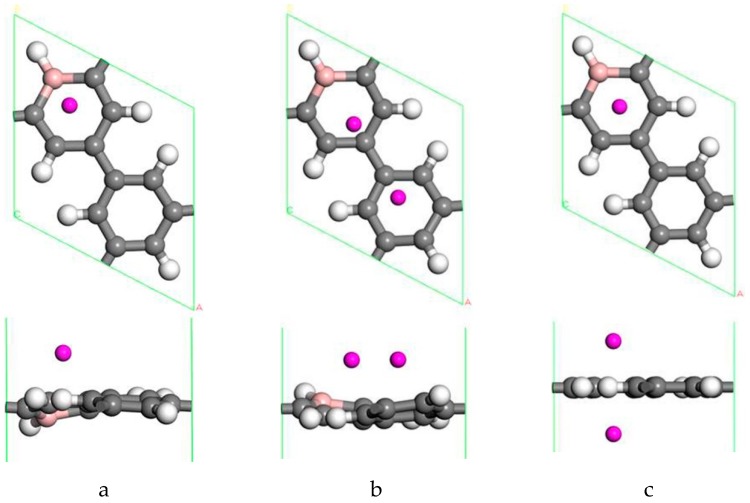
Sc-B/PG system optimized geometry: (**a**) Single Sc atom modification; (**b**) two Sc atoms single-sided modification; (**c**) two Sc atoms double-sided modified in the same pore position. Gray, white, pink, and purple spheres represent C, H, B, and Sc atoms, respectively).

First, the adsorption of Sc atoms on porous graphene doped with a B atom was investigated. When only one Sc atom was modified, six different adsorption sites were considered. As shown in Table 1 on 1B/PG, adsorption sites were named 1, 2, 3, 4, 5, and 6, which represented the hole position of the C ring, C-C bridge, half C ring hole position, large hexagon hole position, C top position and B top position. Results indicated that position 1 was the most easily adsorbed position of a single Sc atom on B-PG, which was similar to the situation of the undoped-PG adsorbing Sc atom [42]. The optimized geometry is shown in Figure 2a. The Sc atom was located at the central hole of the Boron carbon six-membered ring and was slightly deviated. Simultaneously, the B-PG plane was slightly deformed, and the adsorption energy of the Sc atom on the B-PG was −4.004 eV. In our previous study, the adsorption energy of a single Sc atom on clean PG was −2.143 eV [42], which was much smaller than that of single Sc on B-PG of −4.004 eV. It can be seen that B doping can enhance the adsorption activity of the system; compared with pure porous graphene, the Sc atom was closer to the substrate and therefore had greater binding energy on the B-doped PG.

**Figure 3:**
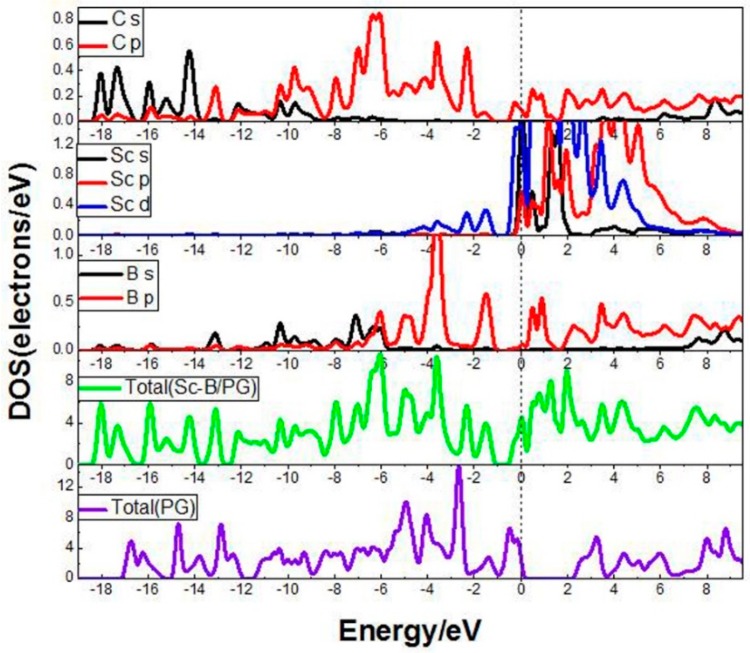
DOS of the Sc-B/PG system and PG system.

Figure 3 shows the partial densities of states (PDOS) of the Sc-B/PG system and the density of states of the clean PG system. It can be seen that the 2p orbit of the B atom and the 3d orbit of the Sc atom overlaps in the interval of −2.0~−1.0 eV, indicating that there was a strong interaction between the B atom and Sc atom. The 2p orbit of the C atom overlaps with the 3d orbit of the Sc atom in the range of −3.0~−1.0 eV. In the interval of −1.0 to 0.0 eV, the peaks of the orbits of C and Sc atoms also overlap, meaning that the C and Sc atoms have an existing strong interaction. In addition, comparing the total density of states of the Sc-B/PG system with the clean PG system, we can see that the 3d orbit of the Sc atom significantly changes the properties of the clean porous graphene near the Fermi surface; the peak of the Sc-B/PG system shifts to the left; and the electrons are close to the Fermi level, the system is more stable after doping the Sc and B atom.

#### 3.2.2. The Adsorption of H_2_ on a Single Sc Atom Modified B-PG System

**Figure 4:**
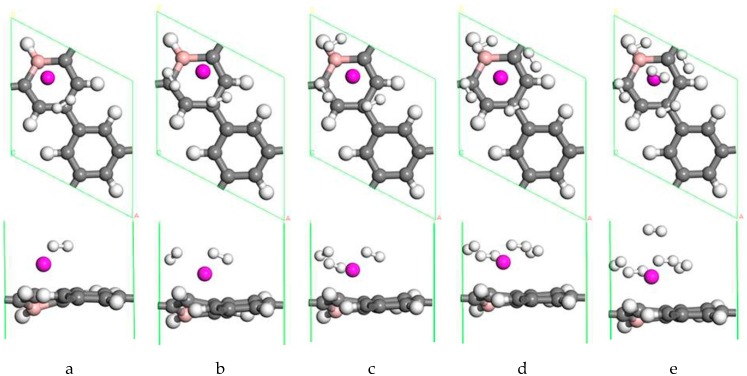
The optimized geometries of a single Sc-modified B-PG system with one H_2_ molecule (**a**), two H_2_ molecules (**b**), three H_2_ molecules (**c**), four H_2_ molecules (**d**) and five H_2_ molecules (**e**) adsorbed.

A single Sc-modified B-doped porous graphene system can adsorb 5 H_2_ molecules, and the optimized structures are given in Figure 4. Moreover, Table 2 lists the adsorption and average adsorption energy of H_2_, the distance between Sc atom and H_2_ molecules, and the distance between the Sc and C atom on the B-PG. A single H_2_ molecule has multiple adsorption sites on Sc-modified B-PG, including the C-C bridge position, the C-H bridge position, the C atom top position, and the Sc atom top position. It has been discovered that the most stable adsorption position of the first H_2_ molecule is the top position of the C atom. This H_2_ molecule has an adsorption energy of −0.677 eV. Meanwhile, the H-H bond was stretched to 0.868 Å. To further explore the hydrogen storage behavior of the system, H_2_ molecules were continuously added to the system. Table 2 indicates that H_2_ molecules’ binding energies fluctuated with the number of adsorbed H_2_ molecules. The third H_2_ molecule adsorbed above the B-H bond and was slightly away from the hexagonal ring, so the adsorption energy decreased suddenly. However, the trend of $d_{Sc-C}$ was a complete contrast to the trend of the H_2_ molecule adsorption energy. That is, the smaller the distance between Sc and the B-PG substrate, the greater the adsorption energy of hydrogen, and vice versa because the addition of hydrogen affects the position of Sc, and the position of Sc in turn affects the adsorption of hydrogen. The system eventually reaches a steady state through this mutually adjusted process. As a result of the symmetry of the H_2_ molecular bonding configuration, all H_2_ molecules were symmetrically distributed on every side of the Sc atom when the fourth H_2_ was adsorbed. The distance from the H_2_ molecule to the Sc atom always increased, but when the fourth H_2_ molecule adsorbed, the $d_{Sc-H_{2}}$ suddenly decreased. At this time, the Sc atom was located at the center of the carbon ring doped by the B atom. Additionally, the first four H_2_ molecules were located on the same horizontal plane, which was parallel to the PG layer. Due to the limited space around the Sc atom and the repulsion between the adsorbed H_2_ molecules, the fifth H_2_ molecule moved to the upper layer after relaxation in Figure 4e. Therefore, the fifth H_2_ molecule had a H-H bond length of 0.762 Å, and it had a minimum adsorption energy of −0.187 eV, which was still bigger than −0.093 eV of the Sc-PG system without B doped [42]. The bond length of the adsorbed H_2_ molecule was in the range of 0.762~ 0.831 Å, and no dissociation of the H_2_ molecule was found. Therefore, the B doped PG decorated by Sc was more suitable for hydrogen storage at room temperature, and the practical application prospect was greater. The PG system modified by a single Sc atom can adsorb up to 5 H_2_ molecules due to the doping of B atom. In a single Sc-modified B-doped porous graphene system, the average adsorption energy (−0.515 eV/H_2_) and the hydrogen storage capacity (4.91 wt.%) are better than that of a Ti-PG system (−0.486 eV) [43].

**Figure 5:**
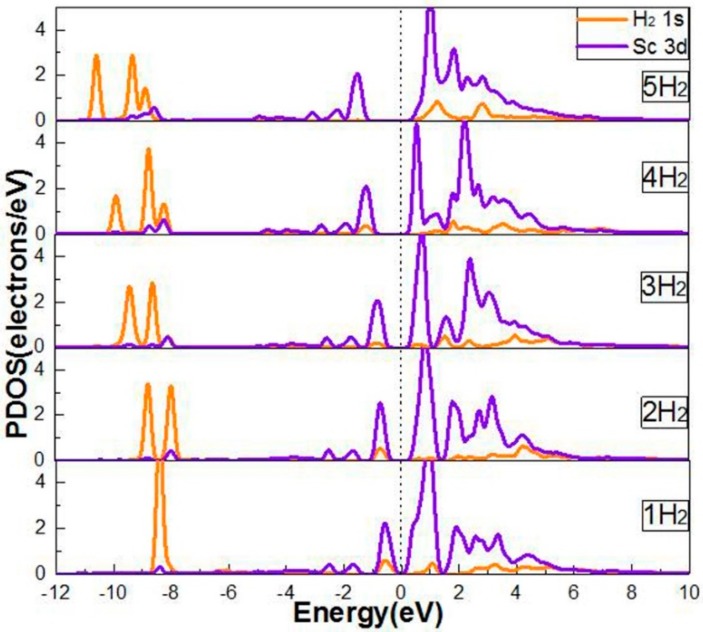
PDOS of Sc-decorated B-PG with one to five H_2_ molecules adsorbed.

Considering the interaction among the Sc atom with the adsorbed H_2_ molecule, we analyzed the partial densities of states (PDOS) of the H_2_ molecule and the Sc atom in Figure 5. It can be seen that the band broadening occurred in the H_2_ molecule from −11.0 to −8.0 eV. In the interval of −9.5~−7.5 eV, the 1s orbit of H_2_ overlapped with the 3d orbit of Sc, indicating that the 1s orbit of H_2_ and the 3d orbit of Sc had a existing interaction. When 1~4 H_2_ molecules were adsorbed on the Sc-decorated B-PG system, it could be seen that the peak of the H_2_ 1s orbit overlapped with the peak of the Sc 3d orbit in the range of −3.0~0.0 eV, which was in accordance with the strong adsorption energy of the H_2_ molecules (except the fifth hydrogen).

**Figure 6:**
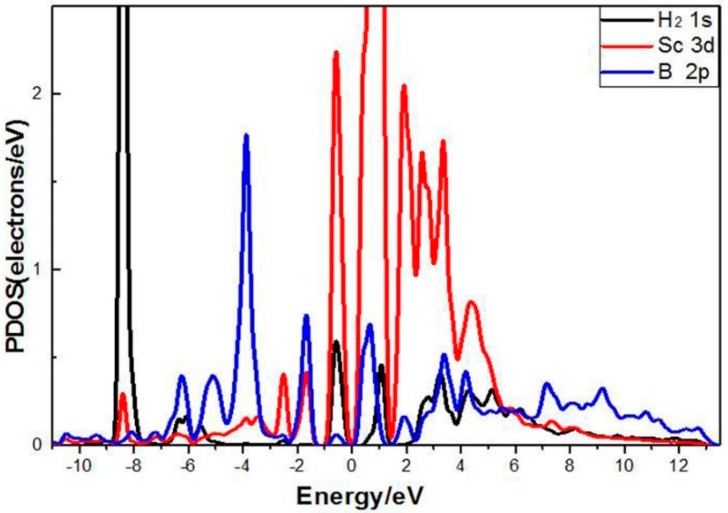
PDOS of Sc-decorated B-PG with one H_2_ molecules adsorbed.

To further investigate the combined effects of the B atom and Sc atom on H_2_ molecule adsorption, the PDOS of one H_2_ molecule adsorbed in a single Sc-modified B-PG system was analyzed (as shown in Figure 6). From 1.0 to 0.0 eV, there were overlapping peaks between B 2p orbit, Sc 3d orbit and 1s orbit of the H_2_ molecule, which means that B and Sc both played a role in H_2_ adsorption. In addition, it can be observed that the 2p orbit of B atoms overlapped with the 3d orbit of Sc atoms in most energy intervals, indicating that there was a strong interaction between B and Sc, which further explained why Sc had a higher binding energy in the B doping system. 

**Figure 7:**
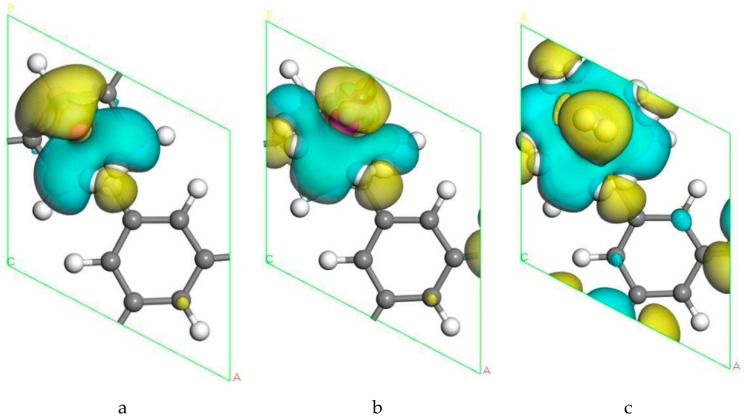
Electronic charge density difference for the Sc-B/PG system in the presence of (**a**) one H_2_ molecule; (**b**) two H_2_ molecules; (**c**) five H_2_ molecules.

The adsorption mechanism could be better understood by analyzing the charge density difference. Figure 7 shows the electronic charge density difference of the system after H_2_ molecules adsorbed. The blue and yellow isosurfaces represented the electron accumulation and electron loss regions, respectively, and the isosurface unit was 0.007 e/${Å}^{3}$. It can be observed from Figure 7a,b that the yellow electron-depleting region was concentrated between the Sc atom and the B-PG, meaning that there was a large amount of charge transfer from the Sc atom to the B-PG. We also analyzed the Mulliken charge population before and after the adsorption of the Sc atom in the B-PG system. We found that the B atom 2p orbit and the C atom had an accumulation of electrons, while the 4s orbit of Sc lost electrons by 1.5 e. Combined with the PDOS of Sc, B and C atom in Figure 3, it was found that the 4s orbit of Sc transferred some electrons to the 2p orbit of B and C, respectively. The charge population indicated that the 3d orbit of Sc obtained 0.45 e electrons, which meant that the B-PG transferred electrons to the Sc atom. On the whole, the Sc carries a positive charge, and the B-PG layer carries a negative charge. Therefore, an electric field is formed between the Sc atom and the B-PG. The adsorption energy of Sc atoms on B-PG was large due to the strong electrostatic Coulomb interaction between Sc and B-PG, and the orbital interaction between Sc, B and C atoms. Since the Sc atom has a positive charge, this will attract the negative charge in H_2_ and accumulate near the metal. It can also be seen from Figure 7 that the blue electron region was concentrated between H_2_ and Sc. Near the side of the Sc atom, polarization occurred in the perpendicular direction of the H_2_ molecule. It has been indicated that the H_2_ molecule undergoes charge redistribution due to the electrostatic field between the Sc atom and the B-PG. Combined with the Mulliken charge population of the 1Sc-B-PG system it adsorbed two H_2_ molecules, the H atoms in the polarized H_2_ molecule were negatively charged at −0.17 e and −0.16 e. Conversely, the Sc atom was positively charged at 1.94 e. Therefore, there was a Coulomb attraction between the H_2_ molecule and the Sc atom. The C atom also had a partial charge transfer on B-PG, indicating that these C atoms also play a role on the H_2_ molecule adsorption. Consequently, the adsorption of H_2_ molecules in the Sc-modified B-PG system is mainly attributed to the orbital interaction between H and Sc atoms. Furthermore, the Coulomb attraction between H_2_ molecules (negatively charged) and Sc atoms (positively charged) strengthen the adsorption of H_2_ molecules.

To better understand the bonding environment of all B-H bonds in this structure after adsorption, we calculated a Bond Population of B-H bond and Mulliken charge Population of B, H, and Sc atoms in B-PG, Sc-B/PG and H_2_ adsorbed Sc-B/PG system, where H was the H atom in the first to fifth hydrogen molecules adsorbed. As shown in Table 3, the B-H population gradually increased as the hydrogen molecules continued to adsorb. After Sc atom modification, the positive charge of B atom decreased rapidly, indicating that Sc was stable on B-PG. Thereafter, the positive charge of the B atom fluctuated with the adsorption of hydrogen. The positive charge of Sc in the hydrogen storage process had been increasing. After the first H_2_ molecule was adsorbed by the Sc-B/PG structure, the Sc atom was charged to 1.72 e, which is greater than that of 1.56 e in the undoped B atom system [42]. It was shown that after doping the B atom, the catalytic effect of Sc atoms in hydrogen storage was more obvious. As the hydrogen molecules continuously adsorbed, the H atom in H_2_ molecules started to be negatively charged and the charge amount gradually decreased. The H atom of the first H_2_ molecule after doping B atoms was charged −0.17 e, and its absolute value was greater than that of −0.13 e in the undoped system [42]. This indicated that doping B atom enhanced the interaction between H_2_, Sc atoms and PG, which was more conducive to hydrogen storage. 

### 3.3. Two Sc Atoms Modified B-PG System

#### 3.3.1. Adsorption Structure of two Sc Atoms Modified B-PG

Transition metal atoms are prone to aggregation on the surface of graphene, resulting in a decrease in the free surface area of the graphene. Metal aggregation is mainly attributed to the high cohesive energy of the atoms [44,45,46]. The cohesive energy of the Sc atom is 3.900 eV [47], each Sc atom is equivalent to an H_2_ adsorption site. To further increase the amount of hydrogen storage, a second Sc atom is added to the same side of the system. Figure 2b shows one of the most stable geometries for adsorbing two Sc atoms on B-PG. It can be seen that the second Sc atom tended to adsorb at the center of the other carbon ring instead of agglomeration with the first Sc atom, which was attributed to the Coulomb repulsion between the two Sc atoms (the two Sc atoms have a positive charge of 0.90 e, 0.97 e, respectively), and there was a strong interaction between the Sc atom and the B-PG. The average binding energy of the two Sc atoms on B-PG was −4.069 eV, and its absolute value was greater than the cohesive energy of the Sc atom, which was 3.900 eV. This means that the adsorption structure of the two Sc atoms on PG is more stable than that of the single Sc atom on B-PG.

The structure of two Sc atoms on both sides of B-PG was also studied to open up enough space to adsorb H_2_ molecules. Each transition metal atom is an active adsorption site. The double-sided modification of B-PG by the transition metal atom can raise the hydrogen storage area. On account of Figure 2a, the second Sc atom had four adsorption sites on the opposite side of the B-PG, including two carbon ring centers, C-C bridge site, and half C ring pore sites on the opposite side. The most stable adsorption structure was obtained as shown in Figure 2c. The average binding energy of two Sc atoms was −4.085 eV, larger than that of two Sc atoms in the clean PG system. Therefore, the presence of B atoms in PG prevented the Sc atoms from aggregating. In addition, it can be seen from Figure 2a,b that the B-PG system underwent slight deformation after Sc atoms were adsorbed. However, Figure 2c did not undergo any deformation, which was related to the symmetric distribution of Sc atoms.

#### 3.3.2. Hydrogen Storage of two Sc Atoms Modified B-PG

**Figure 8:**
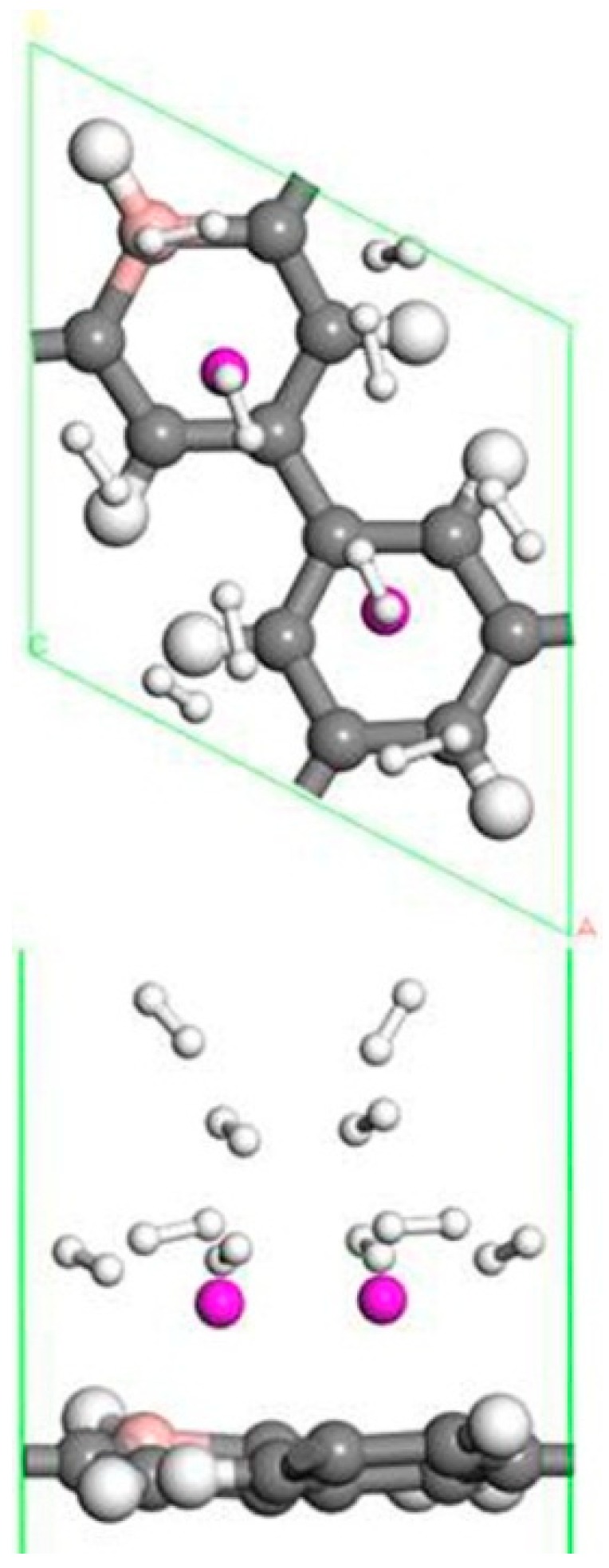
The optimized geometries for ten H_2_ molecules adsorbed on two Sc atoms single-sided modified B-PG.

The B-PG system with two Sc atoms modified on one side can adsorb 10 H_2_ molecules. The optimized geometry structure is shown in Figure 8. The average adsorption energy of the B-PG system was about −0.339 eV/H_2_ with the hydrogen storage capacity (7.73 wt.%). Because the adsorption energy of two Sc atoms on B-PG was larger than their cohesion energy, the distance between them was close without bonding.

**Figure 9:**
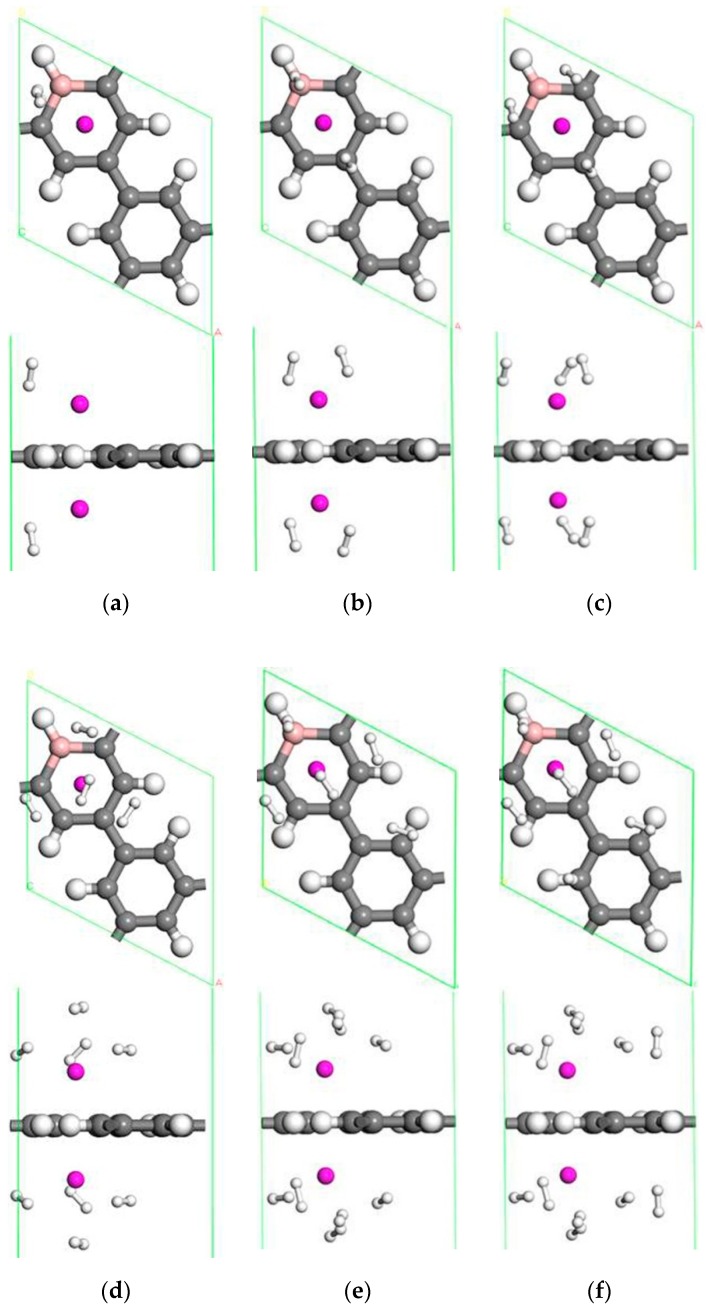
Optimized geometries of two Sc double-sided modified B-PG systems with two H2 molecules (**a**), four H2 molecules (**b**), six H2 molecules (**c**), eight H2 molecules (**d**), ten H2 molecules (**e**), and twelve H2 molecules (**f**) adsorbed.

Figure 2c is a stable structure of two Sc atoms modified B-PG on both sides, and the structure after hydrogen storage is shown in Figure 9. The distance between the two Sc atoms was large enough to prevent the aggregation where double-sided modified B-PG was concerned. There was no deformation that occurred in the B doped porous graphene surface, which was different from the distortion in the single Sc modified B-PG after hydrogen storage. The first eight H_2_ molecules were symmetrically distributed, but the ninth to twelfth H_2_ molecules gradually moved away from the Sc atoms and tended to adsorb to the top of the C-H bond of adjacent carbon rings. Therefore, the average adsorption energy of H_2_ decreased. Two Sc double-sided modified B-PG systems can adsorb up to 12 H_2_ molecules with an average adsorption energy of −0.225 eV/H_2_. According to the standards of the U.S. Department of Energy and the International Energy Agency, the hydrogen storage capacity of ideal hydrogen storage materials should be greater than 5.50 wt.%, and the adsorption energy between H_2_ molecules and materials should be from 0.200 to 0.700 eV [48,49]. Therefore, the average adsorption energy of the H_2_ molecule was also in the range of reversible hydrogen storage. The corresponding theoretical hydrogen storage capacity is 9.13 wt.%, which is higher than the storage hydrogen content of the Sc-PG system 9.09 wt.% [42] and is greater than the amount of hydrogen storage of the Y-PG system 7.87 wt.% [36]. The interaction between the H_2_ molecule, the Sc atom and the B-PG system in the two Sc atoms modified the B-PG system and was similar to the single Sc atom modification B-PG system, and is not described here. By comparing Figure 4e, Figure 8 and Figure 9f, it can be seen that the single Sc atom modified B-PG and the two Sc atoms single-sided modified B-PG adsorbed H_2_ molecules were layered. However, there was no hydrogen stratification with two Sc double-sided decorated B-PG. Moreover, the latter two H_2_ molecules adsorbed above the C-H bond of another carbon six-membered ring. Therefore, the hydrogen storage spaces were larger in the Sc double-sided modification B-PG system than the Sc single-sided modification. In addition, the H_2_ molecules around a single Sc were parallel to the B-PG plane. When the two Sc atoms were modified on the same side, the H_2_ molecule changed from parallel to vertical, but they were not completely perpendicular to the B-PG surface due to the interaction. The perpendicular adsorption of H_2_ molecules is more likely to occur when two Sc atoms are modified at double sides, which is due to the interaction of H_2_ molecules on both sides of the B-PG. In summary, the two Sc located in the same boron-carbon ring position on the opposite side of B-PG is the most suitable structure for hydrogen storage, and Sc modified B-PG is a promising hydrogen storage material.

## 4. Conclusions

We unambiguously investigated the hydrogen storage of the B atom doped Sc decorated PG. With an average adsorption energy (−0.515 eV/H_2_), the most stable adsorption position could adsorb five H_2_ molecules on the center of the boron-carbon ring for a single Sc atom system. By adding a second Sc atom to another side of the PG system, the hydrogen storage capacity effectively could be improved. When the two Sc atoms were located at both sides of the central boron-carbon hexagon, the theoretical hydrogen storage capacity could reach the maximum (~9.13 wt.%), which could adsorb twelve H_2_ molecules. At the same time, the two Sc atoms structure was the most suitable hydrogen storage for the Sc-B/PG system, possessing the average adsorption energy (−0.225 eV/H_2_). Furthermore, the adsorption of these H_2_ molecules in the Sc-modified B/PG system was also responsible for two aspects via the analysis of DOS and charge population: (i) The orbital interaction between H and Sc atoms; (ii) the Coulomb attraction between H_2_ molecules (negatively charge) and Sc atoms (positively charge). The H_2_ molecules were negatively charged due to polarization of the electrostatic field between Sc and B-PG. Therefore, Sc-modified B-doped porous graphene is expected to be used in the field of hydrogen storage.

## Figures and Tables

**Table 1:** Optimized geometries, lattice constants, representative B-H bond lengths, and formation energies of the B-doped porous graphene (PG) system. Note: Gray, white and pink balls represent C, H and B atoms, respectively.

	1B/PG	2B/PG	3B/PG	4B/PG	5B/PG	6B/PG
**Top View**	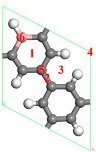	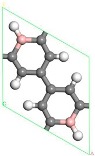	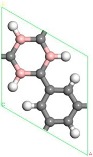	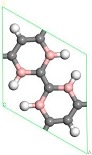	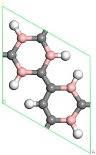	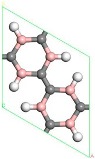
**a (Å)**	7.57	7.62	7.72	7.79	7.88	7.95
$d_{B-H\;}$ **(Å)**	1.19	1.19	1.20	1.19	1.20	1.21
$E_{f}\;$ **(meV/atom)**	121.30	168.03	247.61	291.03	377.73	440.99

**Table 2:** Calculated adsorption energy and average adsorption energy of H_2_, distance between Sc atom and H_2_ molecules, and minimum distance between Sc atom and C atom in Sc-modified B-PG system.

Number of H_2_	1H_2_	2H_2_	3H_2_	4H_2_	5H_2_
$E_{ad}\;$ **(eV)**	−0.677	−0.682	−0.416	−0.614	−0.187
${\bar{E}}_{ad\;}$ **(eV)**	−0.677	−0.680	−0.592	−0.597	−0.515
$d_{Sc-H_{2}}\;$ **(Å)**	2.034	2.049	2.146	2.061	2.867
$d_{Sc-C}\;$ **(Å)**	2.274	2.229	2.380	2.333	2.385

**Table 3:** Calculated Bond Population of the B-H bond and Mulliken Charge Population of B, H, Sc atoms in B-PG, Sc-B/PG and H_2_ adsorbed Sc-B/PG system.

System	B-PG	Sc-B/PG	1H_2_	2H_2_	3H_2_	4H_2_	5H_2_
**B-H**	1.06	1.04	1.03	1.04	1.05	1.05	1.06
**B (e)**	0.33	0.04	0.04	0.03	0.06	0.06	0.05
**Sc (e)**	None	1.15	1.72	1.94	2.18	2.46	2.64
**H (e)**	None	None	−0.17	−0.16	−0.13	−0.16	−0.10
